# Efficacy and safety of robot-assisted deep brain stimulation for Parkinson’s disease: a meta-analysis

**DOI:** 10.3389/fnagi.2024.1419152

**Published:** 2024-05-31

**Authors:** Zhilong Huang, Lian Meng, Xiongjie Bi, Zhengde Xie, Weiming Liang, Jinyu Huang

**Affiliations:** The First Affiliated Hospital of Guangxi University of Science and Technology, Guangxi University of Science and Technology, Liuzhou, Guangxi, China

**Keywords:** Parkinson’s disease, deep brain stimulations, robot, vector error, meta-analysis

## Abstract

**Objective:**

This meta-analysis aims to assess the effectiveness and safety of robot-assisted deep brain stimulation (DBS) surgery for Parkinson’s disease(PD).

**Methods:**

Four databases (Medline, Embase, Web of Science and CENTRAL) were searched from establishment of database to 23 March 2024, for articles studying robot-assisted DBS in patients diagnosed with PD. Meta-analyses of vector error, complication rate, levodopa-equivalent daily dose (LEDD), Unified Parkinson’s Disease Rating Scale (UPDRS), UPDRS II, UPDRS III, and UPDRS IV were performed.

**Results:**

A total of 15 studies were included in this meta-analysis, comprising 732 patients with PD who received robot-assisted DBS. The pooled results revealed that the vector error was measured at 1.09 mm (95% CI: 0.87 to 1.30) in patients with Parkinson’s disease who received robot-assisted DBS. The complication rate was 0.12 (95% CI, 0.03 to 0.24). The reduction in LEDD was 422.31 mg (95% CI: 68.69 to 775.94). The improvement in UPDRS, UPDRS III, and UPDRS IV was 27.36 (95% CI: 8.57 to 46.15), 14.09 (95% CI: 4.67 to 23.52), and 3.54 (95% CI: −2.35 to 9.43), respectively.

**Conclusion:**

Robot-assisted DBS is a reliable and safe approach for treating PD. Robot-assisted DBS provides enhanced accuracy in contrast to conventional frame-based stereotactic techniques. Nevertheless, further investigation is necessary to validate the advantages of robot-assisted DBS in terms of enhancing motor function and decreasing the need for antiparkinsonian medications, in comparison to traditional frame-based stereotactic techniques.

**Clinical trial registration**: PROSPERO(CRD42024529976).

## 1 Introduction

Deep brain stimulation (DBS) for the treatment of Parkinson’s disease (PD) has undergone significant advancements since its inception 30 years ago (Benabid et al., 1987). DBS is an FDA-approved therapy for movement disorders such as Parkinson’s disease, epilepsy obsessive-compulsive disorder, dystonia and essential tremor (Hariz et al., 2002; Herzog et al., 2003; Rodriguez-Oroz et al., 2005; Deuschl et al., 2006; Anheim et al., 2008; Jankovic, 2008; Benabid et al., 2009; Mian et al., 2010; Cury et al., 2017; Lake et al., 2019; Zangiabadi et al., 2019). Studies have demonstrated that it was more effective than medicinal intervention in individuals with Parkinson’s disease and primary motor difficulties (Burchiel et al., 2013; Sato et al., 2019; Paff et al., 2020).

DBS has relied on arc-radius frame-based systems since its creation in 1949 by Leksell. These systems are considered the benchmark for achieving precise and accurate results (Rahman et al., 2009; Khan and Henderson, 2013). Enhancing operational efficiency and precision is crucial consideration in enhancing DBS procedure for movement disorders (Bari et al., 2015). By prioritizing the optimal utilization of operating room and anesthetic time, it is anticipated that patients’ surgical experiences, comfort, and safety will be enhanced (Fenoy and Simpson Jr., 2014; Tolleson et al., 2014). Stereotactic precision is known to be crucial to results in movement disorders, but it can frequently be tedious, error-prone, and time-consuming to achieve acceptable levels (Lanotte et al., 2002; Starr et al., 2004).

With the advent of cutting-edge robotic guiding systems, stereotaxy has undergone a sea change for numerous procedures, such as stereoelectroencephalography (Joseph et al., 2017; Brandmeir et al., 2018). The primary goal of surgical robots is to guarantee and improve the accuracy of a specific operation. With their precise, repeatable, and predefined pathways, robots can safely navigate around obstacles and avoid harming neighboring structures (Davies, 2000). Recent years have seen a rise in the use of robot-assisted procedures for DBS, first in Europe (Lefranc and Le Gars, 2012; Moran et al., 2020), then in Asia (Lefranc and Le Gars, 2012; Liu et al., 2020), and most recently in the US (Vadera et al., 2017; Faraji et al., 2020). Numerous facilities have begun incorporating robotic systems like ROSA (Zimmer Biomet Inc.) and neuromate (Renishaw plc) into their workflow due to the high levels of accuracy and reproducibility that these systems provide (Lefranc and Le Gars, 2012; Liu et al., 2020; Moran et al., 2020).

This study involved a meta-analysis to thoroughly evaluate the available evidence in studies regarding the effectiveness and safety of robot-assisted DBS for PD. The primary outcome was vector error, while the secondary outcomes included complication rate, LEDD, UPDRS, UPDRS II, UPDRS III, and UPDRS IV.

## 2 Materials and methods

### 2.1 Search strategy

The present meta-analysis followed the 2020 guidelines established by the Preferred Reporting Project for Systematic Review and Meta-Analysis (PRISMA; Page et al., 2021). The study has been registered at PROSPERO with the registration number CRD42024529976. A comprehensive search was performed in four databases, including PubMed, Embase, Web of Science, and the Cochrane Library, to retrieve literature published up until March 23, 2024. The search technique adhered to the PICOS principle and utilized a blend of MeSH terms and unrestricted text phrases. The search strategy employed was to combine the terms “Parkinson’s Disease,” “Deep Brain Stimulation” and “robot.” Supplement material 1 offered a thorough summary of the search record.

### 2.2 Inclusion and exclusion criteria

Inclusion criteria: (1) patients diagnosed as idiopathic Parkinson’s Disease; (2) at least one group of patients received robot-assisted DBS; (3) at least one of the following outcomes were reported: vector error, complication rate, levodopa-equivalent daily dose (LEDD), Unified Parkinson’s Disease Rating Scale (UPDRS), UPDRS II, UPDRS III, and UPDRS IV; (4) Types of study was randomized controlled trial, prospective study or retrospective study.

Exclusion criteria: (1) other types of articles, such as case reports, publications, letters, comments, reviews, meta-analyses, editorials, protocols, etc.; (2) other diseases, including secondary Parkinson’s syndrome and atypical Parkinson patients; (3) not robot-assisted surgery; (4) no DBS was performed; (5) duplicate patient cohort; (6) failed to extract data.

### 2.3 Selection of studies

The procedure of selecting literature, which included eliminating duplicate entries, was carried out using EndNote (Version 20; Clarivate Analytics). Two independent reviewers conducted the first search. They removed any duplicate records, evaluated the titles and abstracts to determine their relevance, and classified each study as either included or excluded. We reached a resolution by achieving consensus. In the absence of consensus among the parties, a third reviewer assumed the position of a mediator.

### 2.4 Data extraction

The data was extracted by two reviewers independently. The extracted data included: (1) Basic information of the study, including the first author, publication year, country, study design, sample size, and main outcomes; (2) Baseline characteristics of study subjects, including number of patients, age, disease; (3) The data analyzed included Vector error, complication, LEDD, UPDRS, UPDRS II, UPDRS III, and UPDRS IV. In the absence of consensus between the two independent reviewers, a third reviewer assumed the position of a mediator.

### 2.5 Quality assessment

Two independent reviewers assessed the quality evaluation in the trials that were included. The Newcastle-Ottawa Scale (NOS; Stang, 2010) was utilized to assess the quality of retrospective cohort studies included, while the methodological index for non-randomized studies (MINORS; Slim et al., 2003) for single-arm studies. If there were any discrepancies, the disputed conclusions were resolved through collaborative discussion.

### 2.6 Statistical analysis

The analyses were performed using Stata 12.0. The comparison of continuous variables was performed using the weighted mean difference (WMD) and a 95% confidence interval (CI). The relative ratio (RR) was used to compare binary variables, along with a 95% CI. The medians and interquartile ranges of continuous data were converted to the mean and standard deviation. The Cochrane ‘Sq test and the I^2^ index were used to evaluate the statistical heterogeneity among the studies included (Cumpston et al., 2022). Considering that the papers included in the analysis are sourced from the public literature, it is generally more rational to select the random effect model as the initial choice. A *p*-value below 0.05 was considered to have statistical significance.

## 3 Results

### 3.1 Search results

**Figure 1:**
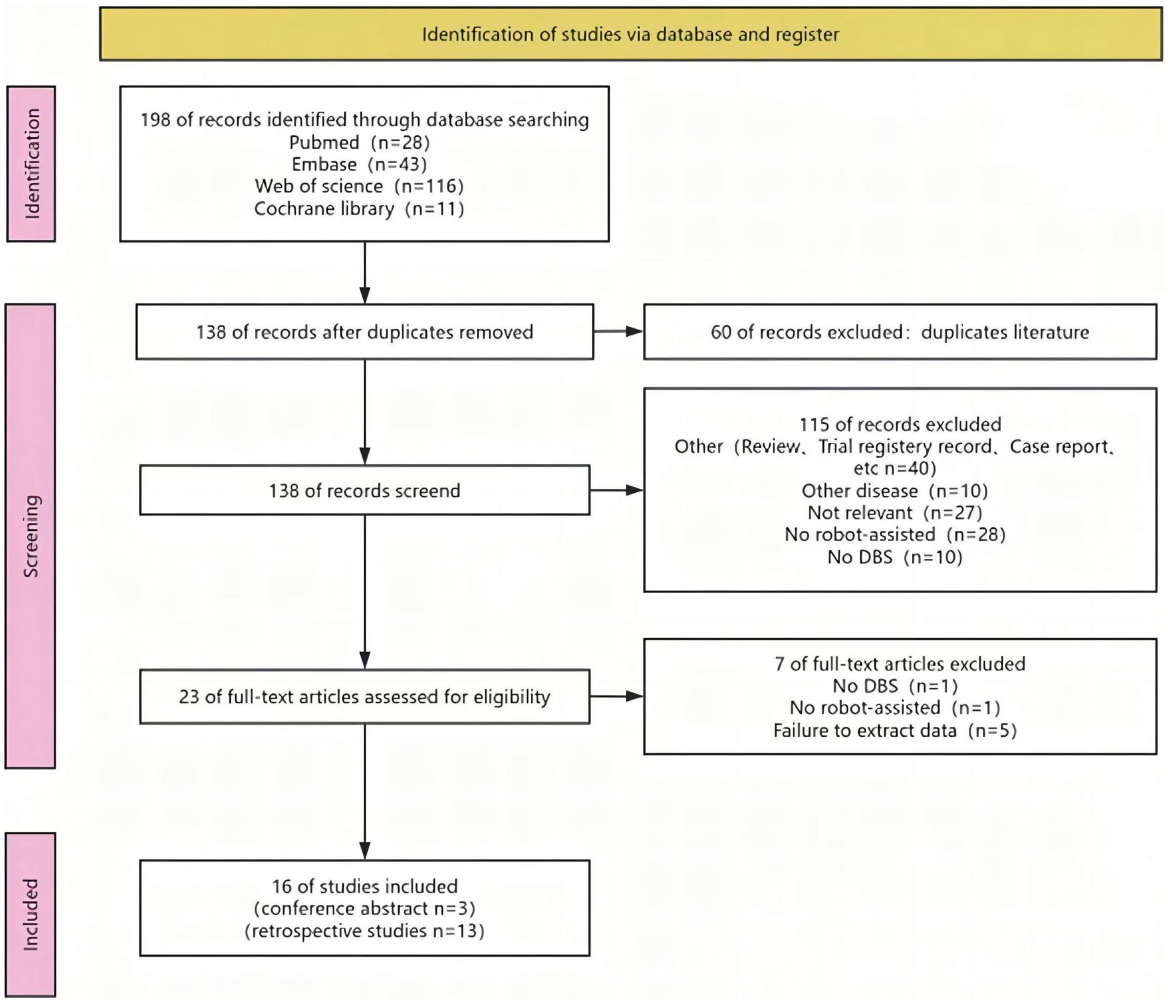
Flow chart of literature search strategies.

Figure 1 depicted the process of selecting and incorporating articles. We initially identified a total of 198 studies. After removing redundant articles, there were only 138 articles left. Upon evaluating the titles and abstracts, a total of 116 publications were determined to be irrelevant and thus excluded. After a comprehensive inspection of the entire text, a total of 16 articles (Delavallee et al., 2016; Lefranc et al., 2017; Ho et al., 2018, 2019; VanSickle et al., 2019; Faraji et al., 2020; Jin et al., 2020; Moran et al., 2020; Paff et al., 2020; Eross and Halasz, 2021; Ribault et al., 2021; Liang et al., 2022; Mei et al., 2022; Soler-Rico et al., 2022; Hegde et al., 2023; Wu et al., 2023) were chosen for inclusion in this meta-analysis.

### 3.2 Patient characteristics and quality assessment

This meta-analysis comprised 15 publications, consisting of six retrospective cohort studies and nine retrospective single-arm studies. The analysis was limited to the data of individuals who underwent robot-assisted DBS for PD, and totally 732 patients were included. The Newcastle-Ottawa Scale (NOS) was utilized to assess the quality of retrospective cohort studies included, while the methodological index for non-randomized studies (MINORS) for single-arm studies. Table 1 presents detailed data on patient characteristics and quality assessment.

**Table 1:** Characteristics of included studies and patients.

Author, year	Country	Study design	Cases	Robot-Assisted Surgery	Age (Mean ± SD)	Male%	Quality
Loránd 2021 (Eross and Halasz, 2021)	Hungary	A	16	ROSA stereotactic robot system	NA	NA	2
Ajay 2023 (Hegde et al., 2023)	UK	A	24	the Renishaw neuromate^®^	60.71 ± 7.3	87.5	4
Allen H 2018 (Ho et al., 2018)	USA	B	30	Mazor’s frameless	NA	NA	4
Maxime 2016 (Delavallee et al., 2016)	Belgium	B	10	Artis Zeego—3D fluoroscopic robotic	57.6 ± 6.5	90.0	12
Amir H 2020 (Faraji et al., 2020)	USA	B	20	Robotic-Assisted Stereotaxy	NA	NA	8
Allen L 2019 (Ho et al., 2019)	USA	B	20	Mazor’s Frameless	67.4	50.0	8
Hai Jin 2020 (Jin et al., 2020)	China	A	153	Leksellstereotactic G frame	63.3 ± 8.2	60.0	7
Michel 2017 (Lefranc et al., 2017)	France	B	23	ROSA^®^ robot	63.0 ± 8.6	60.9	10
Allison S 2022 (Liang et al., 2022)	USA	B	35	Mazor Robot–Assisted Frameless	61.0 ± 14.3	74.0	8
Catherine 2020 (Moran et al., 2020)	Ireland	B	152	the Neuro|Mate^TM^ Robot	60.0 ± 9.0	NA	12
Michelle 2019 (Paff et al., 2020)	USA	A	27	ROSA robot	63.5 ± 11.0	60.0	10
Shams 2021 (Ribault et al., 2021)	France	A	20	RAS Neuromate^®^	62.5 ± 10.0	40.0	8
Morgane 2022 (Soler-Rico et al., 2022)	Belgium	B	32	stereotactic peroperative robotic	60.8 ± 10.3	68.8	10
VanSickle 2019 (VanSickle et al., 2019)	USA	B	128	Mazor Robotics	64.6 ± 13.2	37.5	8
Wu Weidong 2023 (Wu et al., 2023)	China	B	25	neurosurgical robot-assisted	78.3 ± 3.2	56.0	12
Shanshan Mei 2022 (Mei et al., 2022)	China	A	17	frameless robot-assisted Sinovation SR1	57.8 ± 11.8	41.1	5

A: Retrospective cohort study. B: Retrospective single-arm study.

### 3.3 Clinical outcomes

The meta-analysis results for clinical outcomes were consolidated and shown in Table 2.

**Table 2:** The results of the meta-analysis.

Outcomes	No. of study	Patients	Heterogeneity	Overall effect size	95% CI of overall effect
			I^2^(%) *p*-value		
Vector error	10	601	99.00 0.00	1.09	0.87–1.30
Complication rate	12	655	92.88 0.00	0.12	0.03–0.24
Reduction in LEDD	8	435	0.00 0.99	422.31	68.69–775.94
Improvement inUPDRS	3	330	0.00 0.807	27.36	8.57–46.15
Improvement inUPDRS III	5	385	0.00 0.984	14.09	4.67–23.52
Improvement inUPDRS IV	2	175	0.00 0.989	3.54	−2.35-9.43

#### 3.3.1 Vector error

**Figure 2:**
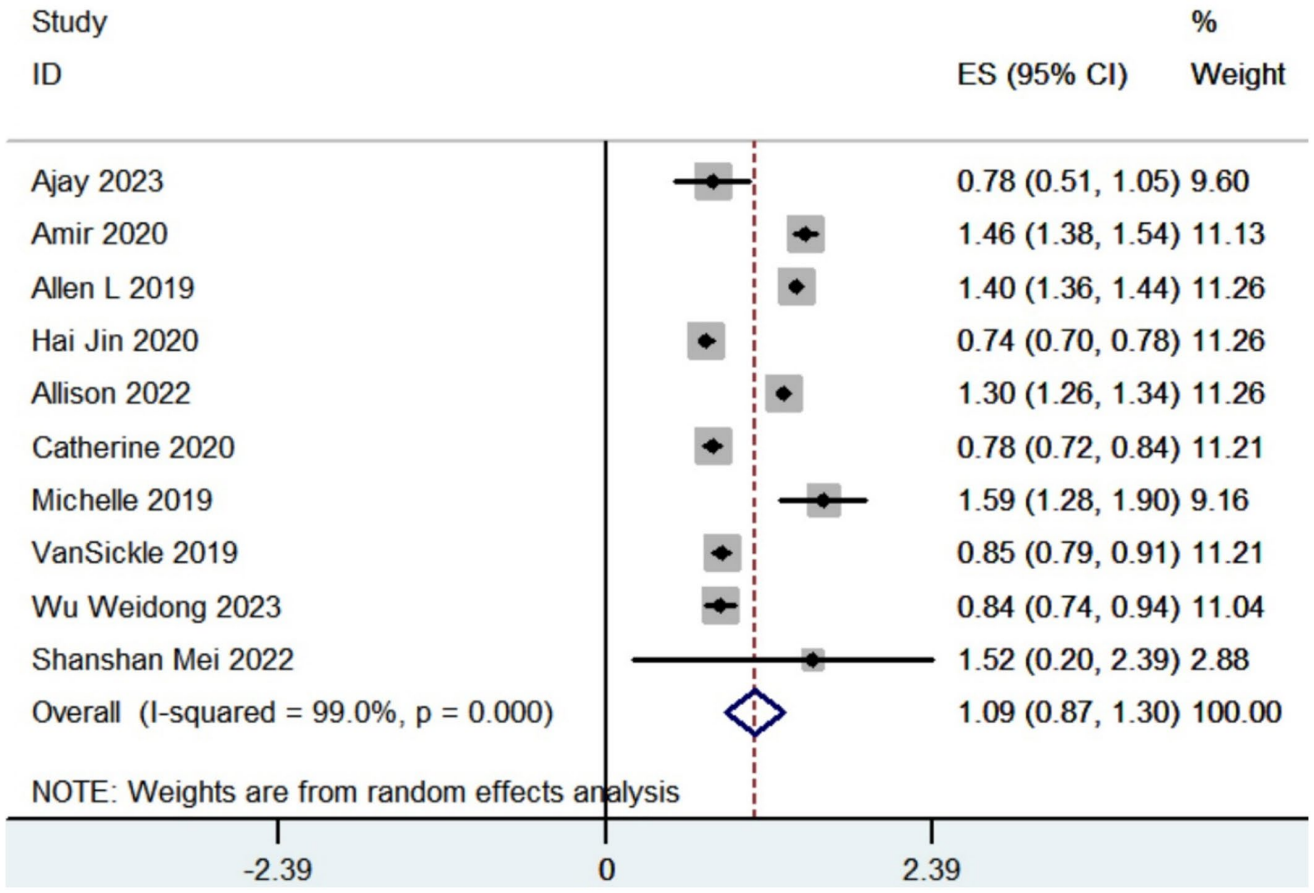
Forest plot of the meta-analysis for vector error.

Totally 10 studies reported vector error. The pooled results revealed that the vector error was measured at 1.09 mm (95% CI, 0.87 to 1.30; Figure 2) in patients with Parkinson’s disease who received robot-assisted DBS.

#### 3.3.2 Complication rate

**Figure 3:**
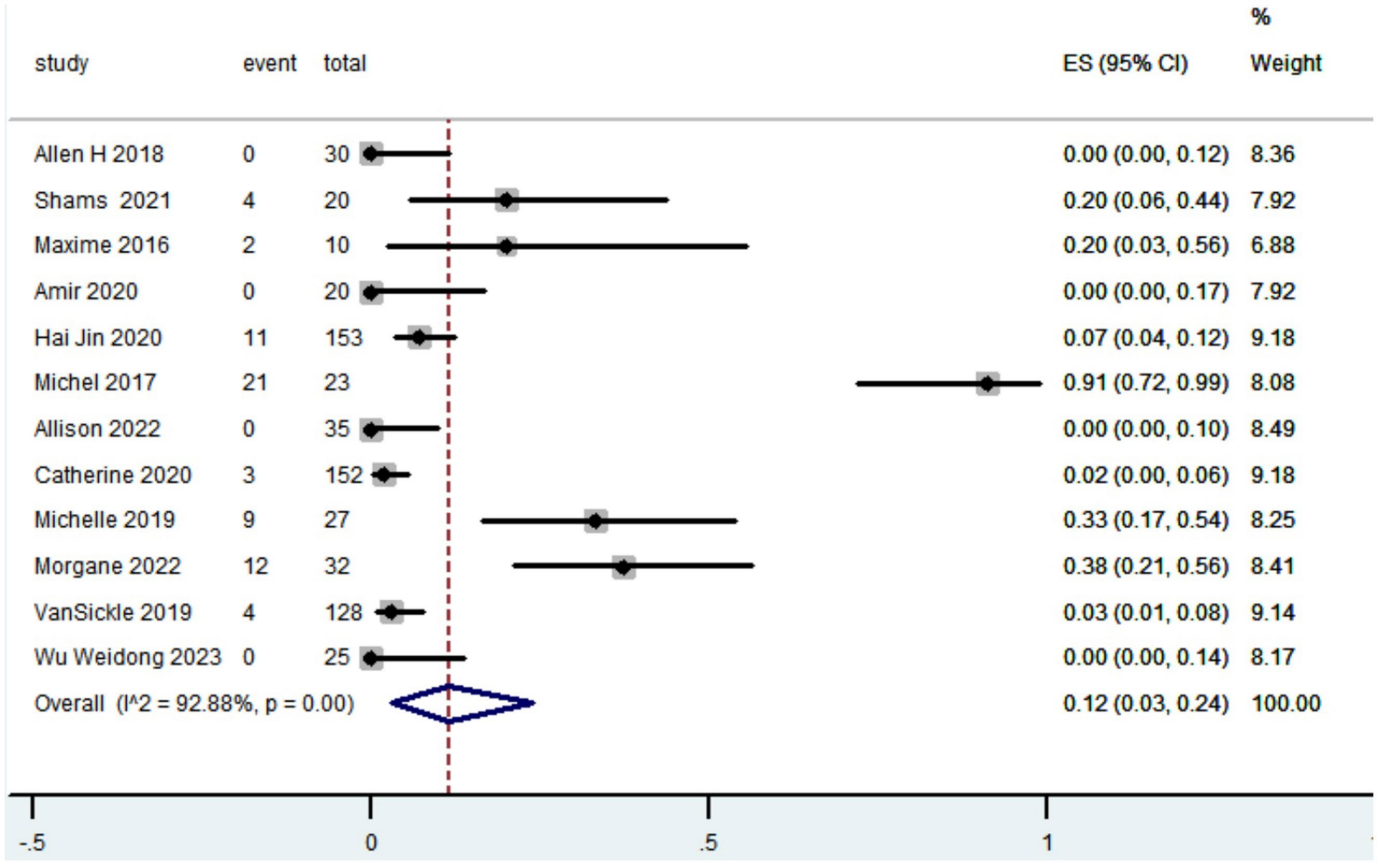
Forest plot of the meta-analysis for complication rate.

Totally 12 studies reported adverse events. The pooled results revealed that the complication rate in patients with Parkinson’s disease who received robot-assisted DBS was 0.12 (95% CI, 0.03 to 0.24; Figure 3). Common complications included hemorrhage, infection and transient confusion.

#### 3.3.3 LEDD

**Figure 4:**
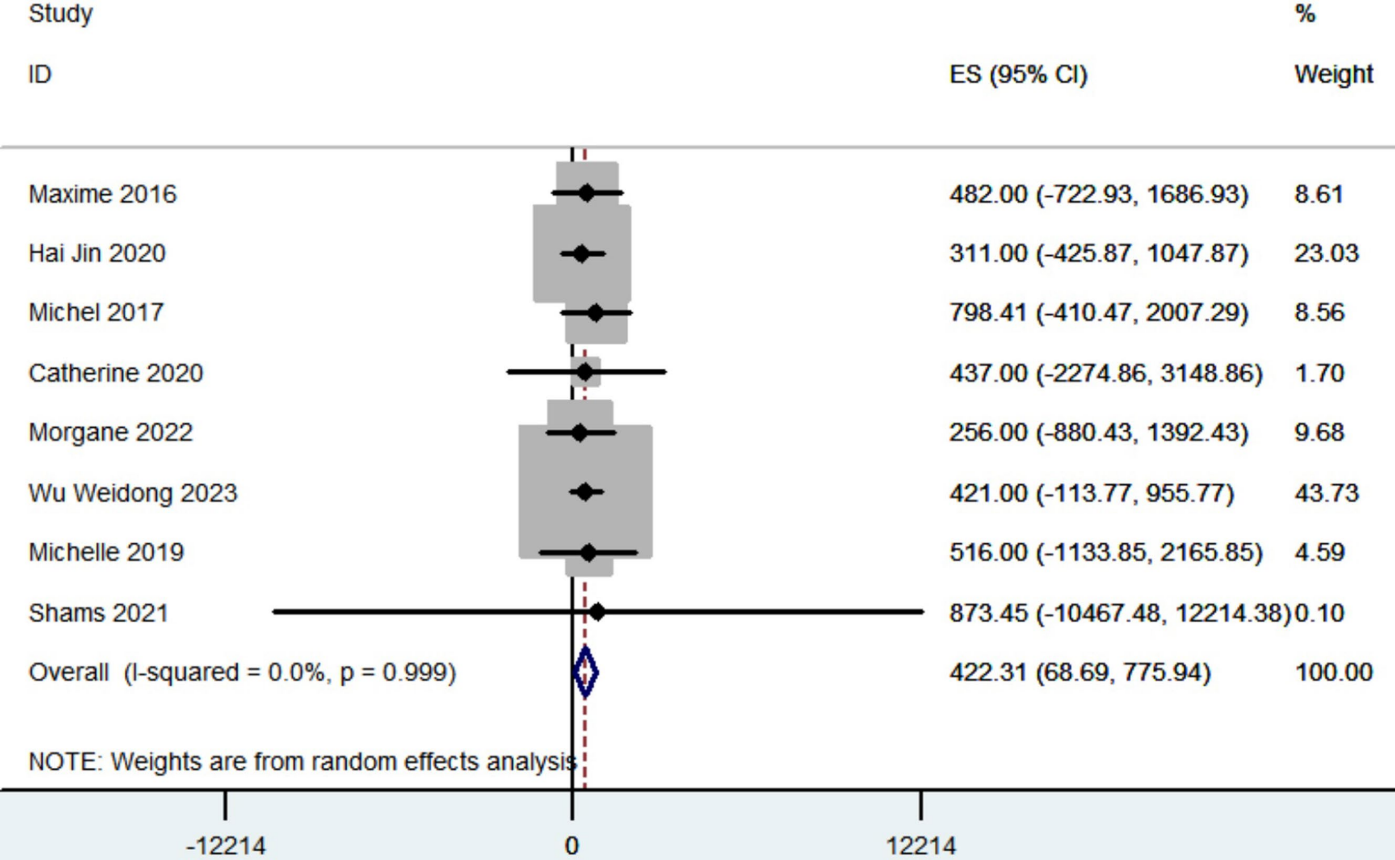
Forest plot of the meta-analysis for reduction in LEDD.

A total of eight articles recorded the reduction in LEDD. The pooled results showed that the reduction in LEDD was 422.31 mg (95% CI: 68.69 to 775.94; Figure 4) after patients with PD received robot-assisted DBS.

#### 3.3.4 UPDRS, UPDRS III, and UPDRS IV

**Figure 5:**
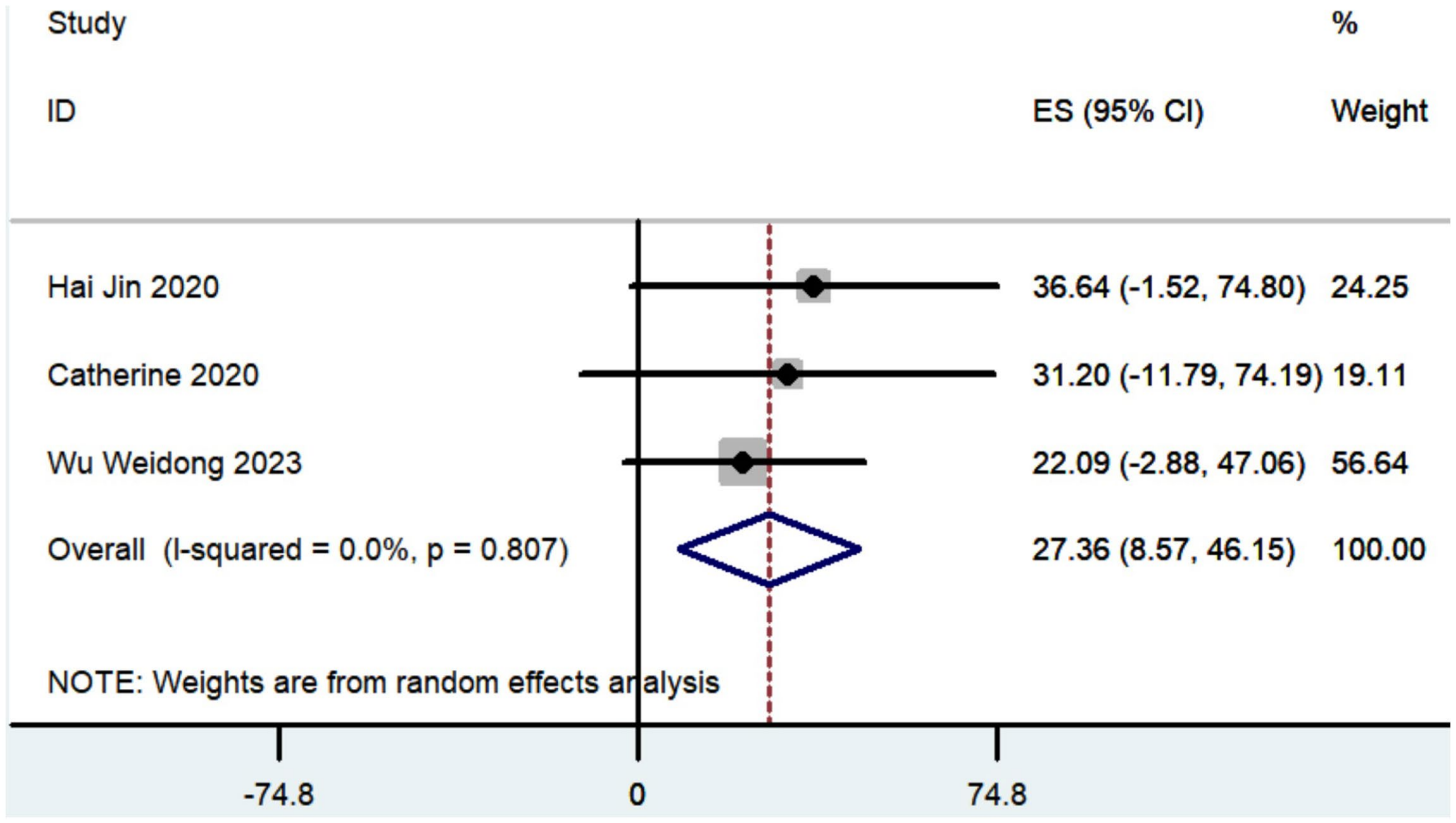
Forest plot of the meta-analysis for improvement in UPDRS.

**Figure 6:**
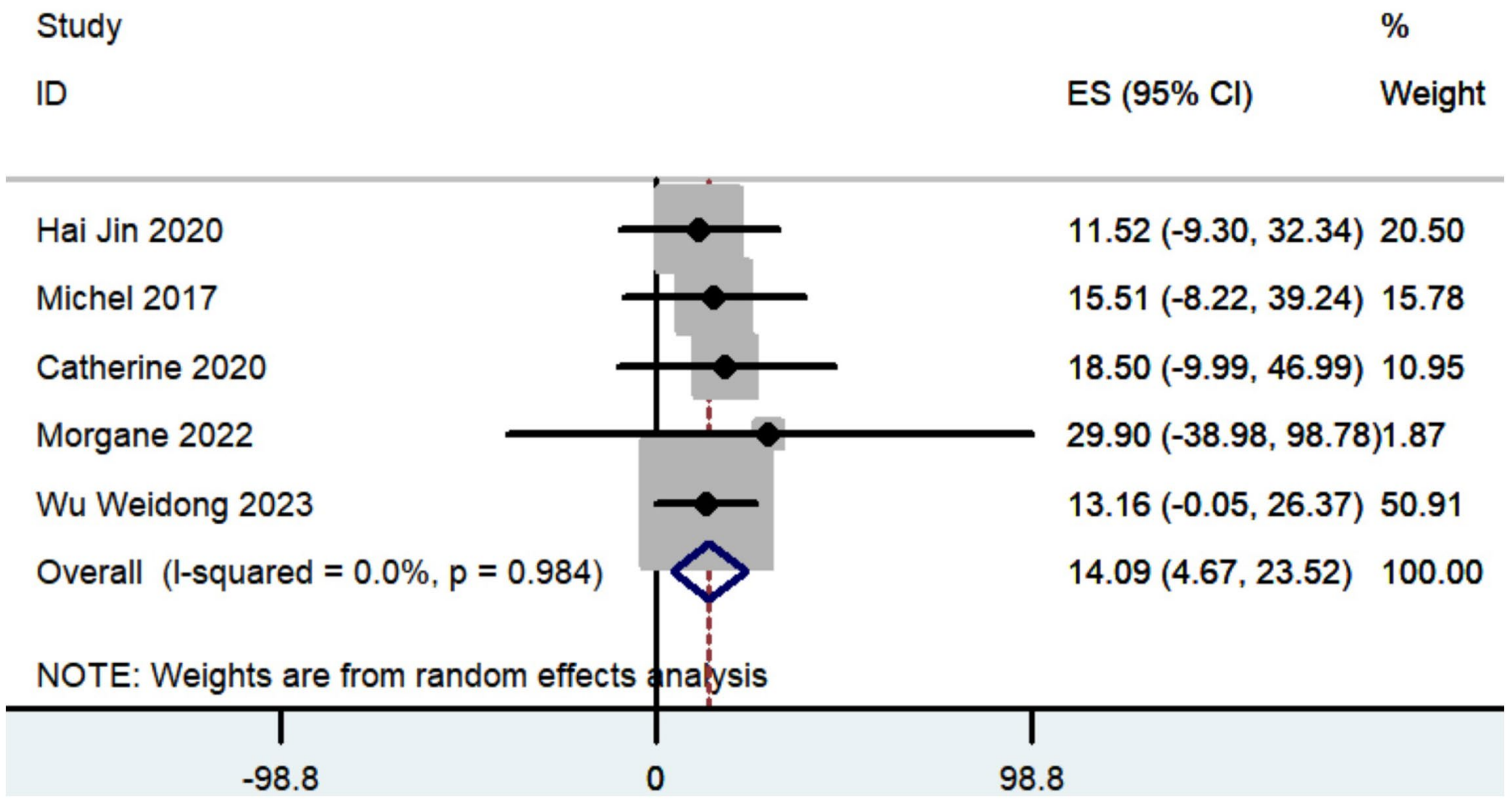
Forest plot of the meta-analysis for improvement in UPDRS III.

**Figure 7:**
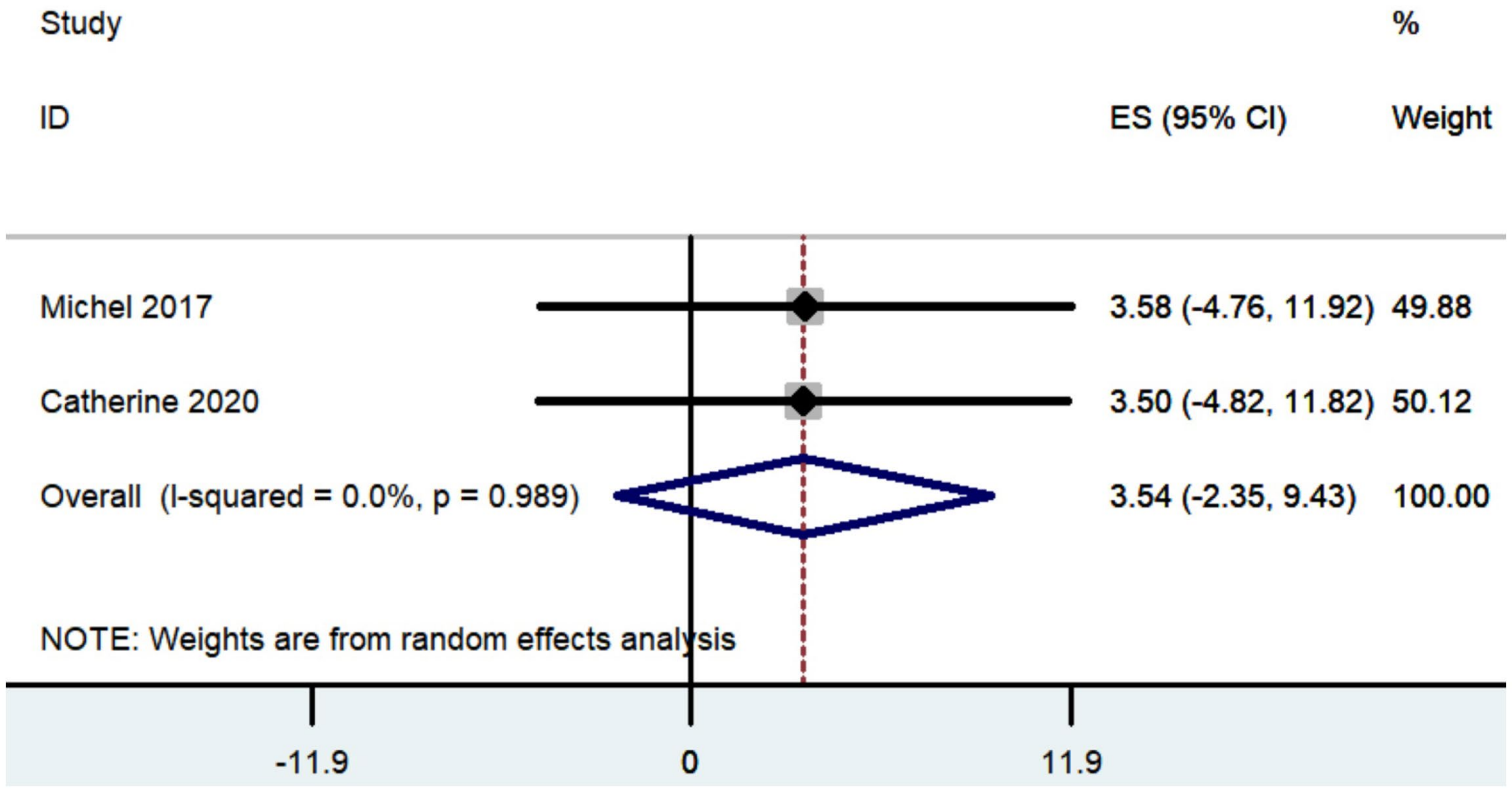
Forest plot of the meta-analysis for improvement in UPDRS IV.

Three studies documented the UPDRS, while five research provided data on UPDRS III, and only two studies included information on UPDRS IV. The aggregated findings demonstrated that the improvement in UPDRS, UPDRS III, and UPDRS IV was 27.36 (95% CI: 8.57 to 46.15; Figure 5), 14.09 (95% CI: 4.67 to 23.52; Figure 6), and 3.54 (95% CI: −2.35 to 9.43; Figure 7), respectively.

## 4 Discussion

Since its initial clinical description in 1995, DBS has been shown to be an effective treatment in multiple randomized controlled trials for patients with Parkinson’s disease who experienced motor fluctuations. This therapy improved severe periods of reduced movement (akinesia) during off-periods and reduced troublesome involuntary movements (dyskinesia) during on-periods (Limousin et al., 1995; Krack et al., 2003). Randomized controlled trial results, with quality of life as the key outcome, have conclusively demonstrated the advantages of neurostimulation over a medically treated control group that did not get stimulation (Deuschl et al., 2006). A recent meta-analysis of five randomized controlled studies comparing STN-DBS to the best available medical treatment has proven that neurostimulation is more effective than the best medical treatment (Krack et al., 2019). The study found a significant improvement of 35.4% in motor symptoms, as judged by the Unified Parkinson’s Disease Rating Scale Part 3 in the absence of medication. Additionally, there was a reduction of 50.8% in off-time and a 49.1% reduction in levodopa-induced dyskinesias. Maximizing operational efficiency and precision continue to be crucial elements in enhancing DBS surgery for movement disorders (Bari et al., 2015). The advancement of contemporary robotic-guidance systems has brought about a significant transformation in stereotaxy, particularly in procedures such as stereoelectroencephalography (Joseph et al., 2017; Brandmeir et al., 2018). Currently, there is significant interest in the use of robot-assisted brain pacemaker surgery for the treatment of Parkinson’s disease. The present meta-analysis aimed to thoroughly evaluate the available evidence in studies regarding the effectiveness and safety of robot-assisted DBS for PD. Since most of studies included were retrospective single-arm studies (Table 1), a single-arm meta-analysis limited to robot-assisted DBS was performed.

Certain centers considered 3 mm to be the benchmark for reimplanting leads, whereas most studies deemed an accuracy of less than 2 mm to be desirable for leads placement (McClelland 3rd et al., 2005; Burchiel et al., 2013). Holl et al. found that if the lead deviates more than 2 mm from the intended nuclei, it can result in reduced therapeutic effectiveness (Holl et al., 2010). Previous studies reported that the vector error was quantified at 1.11 mm to 3.70 mm (Lanotte et al., 2002; Starr et al., 2004; Hamid et al., 2005; Holloway et al., 2005; Bjartmarz and Rehncrona, 2007; Kelman et al., 2010; Starr et al., 2010; Burchiel et al., 2013; Khan and Henderson, 2013; Stieglitz et al., 2013; Sharma et al., 2014; Bot et al., 2015; von Langsdorff et al., 2015; Matias et al., 2018; Neudorfer et al., 2018; Qiu, 2019; Bezchlibnyk et al., 2020; Mei et al., 2022; Chuang et al., 2023; Hegde et al., 2023; Li, 2023; Schulder et al., 2023) when DBS was performed by conventional frame-based stereotactic methods. The vector error was quantified at 1.09 mm in individuals with PD who underwent robot-assisted DBS, according to our findings. Our findings suggest that the precision of robot-assisted DBS appears to surpass that of the traditional frame-based approach. Over time, the precision and dependability of metallic devices used for lead implantation might be affected by wear and deformation. Robot systems are easier to maintain than traditional frame-based systems. The frame-based approach necessitates the repeated autoclaving of the following items following each surgery: the head frame, screws, phantom base, microdriver, and targeting bow. Though the robot does not come into direct touch with the patient during surgery, only a few of components like screws, microdrivers, and instrument holders require autoclaving in the robot system. The frame-based group may have had inferior precision due to deformation, wear, and dullness caused by the mechanical components’ prolonged exposure to heat and accidental bumps. The robot system may be able to keep a better level of precision and accuracy for lead implantations than the frame-based system, but only with long-term, thoughtful and dependable maintenance (Mei et al., 2022). DBS robot-assisted surgery achieves high precision by the implementation of a redesigned registration process, intraoperative registration, and simulated target verification (Xu et al., 2018; Gong et al., 2020; Jin et al., 2020).

In relation to safety, the findings of our study indicate that the complication rate associated with robot-assisted DBS was 0.12 (95% CI, 0.03 to 0.24). The most frequently seen problems included bleeding, infection, and transitory disorientation. Prior research has indicated that the incidence of complications associated with traditional frame-based stereotactic techniques ranged from 0.00 to 0.28 (Herzog et al., 2003; Starr et al., 2004; Rodriguez-Oroz et al., 2005; Deuschl et al., 2006; Weaver et al., 2009; Starr et al., 2010; Keller, 2013; Ostrem et al., 2013; Fenoy and Simpson Jr., 2014; Matias et al., 2018; Qiu, 2019; Paff et al., 2020; Holewijn et al., 2021; Ribault et al., 2021). Robot-assisted DBS did not provide any additional safety concerns as compared to traditional frame-based stereotactic techniques.

The combined findings indicated that patients with PD saw a decrease in LEDD of 422.31 mg (95% CI: 68.69 to 775.94) following the administration of robot-assisted DBS. Previous studies have shown that the LEDD linked to conventional frame-based stereotactic methods ranged from 296 to 900 mg (Herzog et al., 2003; Rodriguez-Oroz et al., 2005; Anheim et al., 2008; Weaver et al., 2009; Fasano et al., 2010; Keller, 2013; Ostrem et al., 2013; Cheng et al., 2014; Bezchlibnyk et al., 2020; Paff et al., 2020; Zhang, 2020; Holewijn et al., 2021; Ribault et al., 2021; Li, 2023). The comparable outcomes observed between robot-assisted DBS and the conventional frame-based stereotactic technique suggest that the LEDD is not influenced by the specific surgical approach employed.

The aggregated findings demonstrated that the improvement in UPDRS, UPDRS III, and UPDRS IV was 27.36, 14.09, and 3.54, respectively. Prior research has demonstrated that traditional frame-based stereotactic procedures resulted in improvements of 12.3 to 50 in UPDRS, 3.2 to 37.9 in UPDRS III, and 3.4 to 44.4 in UPDRS IV (Weaver et al., 2009; Fasano et al., 2010; Keller, 2013; Ostrem et al., 2013; Cheng et al., 2014; Bezchlibnyk et al., 2020; Paff et al., 2020; Zhang, 2020; Holewijn et al., 2021; Ribault et al., 2021; Li, 2023). Our study indicates that the increased accuracy of robot-assisted DBS does not provide substantial advantages over traditional frame-based stereotactic procedures in terms of motor improvement. However, the conclusion drawn may not be entirely accurate due to the limited number of studies that reported findings on motor improvement (Table 2).

While the existing evidence does not disprove the benefits of robot-assisted DBS in terms of motor improvement and reduction of antiparkinsonian drugs, it still provides several advantages (Mei et al., 2022). These include increased patient comfort without the need for a heavy frame, shorter operation time, consistent and reliable positioning of the arm along a specific path, facilitating easier adjustments, and eliminating the manual setup of coordinates. Furthermore, the use of robotic approach is constrained by several restrictions, such as its exorbitant expense, sluggish acceptance and popularization, the necessity of a substantial team, and a protracted learning curve. Nevertheless, the financial strain on both doctors and patients could be alleviated by reducing the duration of surgeries and streamlining the surgical procedure. On the other hand, the frame-based technology poses challenges for the patient due to its extensive processes and extended operation periods, which can result in increased costs.

The advantages of our research are evident. This study is the inaugural meta-analysis to assess the effectiveness and safety of robot-assisted DBS surgery for Parkinson’s disease. Our findings can offer empirical medical support for the utilization of robot-assisted DBS in the management of individuals diagnosed with Parkinson’s disease. However, it is indisputable that our study has certain limitations. At first, because most research were single-arm studies, a single-arm meta-analysis was undertaken particularly on robot-assisted DBS. Consequently, there was a deficiency in directly comparing robot-assisted DBS with normal frame-based stereotactic treatments. Furthermore, all of the studies included in the analysis were retrospective in nature, which increases their susceptibility to bias. In addition, several factors may cause notable differences among the included research’ results. The robotic devices used to perform DBS surgery were different among included studies (Table 1). The study designs varied, with some studies being single-arm studies while others were controlled cohort studies. There were disparities in the basic characteristics of the patients, including their age, gender, LEDD, and UPDRS scores prior to the surgical procedure. The surgical techniques employed by surgeons varied across various medical centers.

In conclusion, our research findings indicate that robot-assisted DBS is a viable and secure method for treating PD. Robot-assisted DBS offers improved precision compared to traditional frame-based stereotactic procedures, which deserves further promotion in clinical application. However, the evidence gathered indicates that there are no significant benefits of robot-assisted DBS in terms of motor improvement and reduction of antiparkinsonian drugs, compared to standard frame-based stereotactic procedures. Therefore, to ensure further verification of the safety and efficacy of robot-assisted DBS, it is crucial to carry out additional multicenter, randomized controlled trials that compare robot-assisted DBS with standard frame-based stereotactic procedures.

## Data availability statement

The datasets presented in this study can be found in online repositories. The names of the repository/repositories and accession number (s) can be found in the article/Supplementary material.

## Author contributions

ZH: Conceptualization, Formal analysis, Investigation, Writing – original draft. LM: Conceptualization, Formal analysis, Investigation, Writing – original draft. XB: Data curation, Supervision, Validation, Writing – original draft. ZX: Data curation, Supervision, Validation, Writing – original draft. WL: Funding acquisition, Resources, Writing – review & editing. JH: Funding acquisition, Resources, Writing – review & editing.

## References

[ref1] Anheim M. Batir A. Fraix V. Silem M. Chabardès S. Seigneuret E. . (2008). Improvement in Parkinson disease by subthalamic nucleus stimulation based on electrode placement: effects of Reimplantation. Arch. Neurol. 65, 612–616. doi: 10.1001/archneur.65.5.612 18474736

[ref2] Bari A. A. Fasano A. Munhoz R. P. Lozano A. M. (2015). Improving outcomes of subthalamic nucleus deep brain stimulation in Parkinson's disease. Expert. Rev. Neurother. 15, 1151–1160. doi: 10.1586/14737175.2015.1081815 26377740

[ref3] Benabid A. L. Chabardes S. Mitrofanis J. Pollak P. (2009). Deep brain stimulation of the subthalamic nucleus for the treatment of Parkinson's disease. Lancet Neurol. 8, 67–81. doi: 10.1016/s1474-4422(08)70291-6 19081516

[ref4] Benabid A. L. Pollak P. Louveau A. Henry S. de Rougemont J. (1987). Combined (Thalamotomy and stimulation) stereotactic surgery of the vim thalamic nucleus for bilateral Parkinson disease. Appl. Neurophysiol. 50, 344–346. doi: 10.1159/000100803, PMID: 3329873

[ref5] Bezchlibnyk Y. B. Sharma V. D. Naik K. B. Isbaine F. Gale J. T. Cheng J. . (2020). Clinical outcomes of Globus pallidus deep brain stimulation for Parkinson disease: a comparison of intraoperative Mri- and Mer-guided Lead placement. J. Neurosurg. 134, 1072–1082. doi: 10.3171/2019.12.jns192010, PMID: 32114534

[ref6] Bjartmarz H. Rehncrona S. (2007). Comparison of accuracy and precision between frame-based and frameless stereotactic navigation for deep brain stimulation electrode implantation. Stereotact. Funct. Neurosurg. 85, 235–242. doi: 10.1159/000103262, PMID: 17534136

[ref7] Bot M. van den Munckhof P. Bakay R. Sierens D. Stebbins G. Verhagen M. L. (2015). Analysis of stereotactic accuracy in patients undergoing deep brain stimulation using Nexframe and the Leksell frame. Stereotact. Funct. Neurosurg. 93, 316–325. doi: 10.1159/000375178, PMID: 26227179

[ref8] Brandmeir N. J. Savaliya S. Rohatgi P. Sather M. (2018). The comparative accuracy of the Rosa stereotactic robot across a wide range of clinical applications and registration techniques. J. Robot. Surg. 12, 157–163. doi: 10.1007/s11701-017-0712-2, PMID: 28484885

[ref9] Burchiel K. J. McCartney S. Lee A. Raslan A. M. (2013). Accuracy of deep brain stimulation electrode placement using intraoperative computed tomography without microelectrode recording. J. Neurosurg. 119, 301–306. doi: 10.3171/2013.4.jns122324, PMID: 23724986

[ref10] Cheng C. Y. Hsing M. T. Chen Y. H. Wu S. L. Sy H. N. Chen C. M. . (2014). Deep brain stimulation for Parkinson's disease using frameless technology. Br. J. Neurosurg. 28, 383–386. doi: 10.3109/02688697.2013.848838, PMID: 24138684

[ref11] Chuang T. C. Tan J. Q. Chen S. M. (2023). Comparison of intraoperative imaging guided versus microelectrode recording guided deep brain stimulation for Parkinson's disease: a Meta-analysis. Neurocirugia (English Edition) 34, 228–237. doi: 10.1016/j.neucie.2022.09.003, PMID: 36931932

[ref12] Cumpston M. S. McKenzie J. E. Welch V. A. Brennan S. E. (2022). Strengthening systematic reviews in public health: guidance in the Cochrane handbook for systematic reviews of interventions, 2nd edition. J. Public Health (Oxf.) 44, e588–e592. doi: 10.1093/pubmed/fdac036, PMID: 35352103 PMC9715291

[ref13] Cury R. G. Fraix V. Castrioto A. Fernandez M. A. P. Krack P. Chabardes S. . (2017). Thalamic deep brain stimulation for tremor in Parkinson disease, essential tremor, and dystonia. Neurology 89, 1416–1423. doi: 10.1212/wnl.0000000000004295, PMID: 28768840

[ref14] Davies B. (2000). A review of robotics in surgery. Proc. Inst. Mech. Eng. H J. Eng. Med. 214, 129–140. doi: 10.1243/0954411001535309, PMID: 10718057

[ref15] Delavallee M. Delaunois J. Ruwet J. Jeanjean A. Raftopoulos C. (2016). Stn Dbs for Parkinson's disease: results from a series of ten consecutive patients implanted under general Anaesthesia with intraoperative use of 3d fluoroscopy to control Lead placement. Acta Neurochir. 158, 1783–1788. doi: 10.1007/s00701-016-2889-y, PMID: 27405941

[ref16] Deuschl G. Schade-Brittinger C. Krack P. Volkmann J. Schäfer H. Bötzel K. . (2006). A randomized trial of deep-brain stimulation for Parkinson's disease. N. Engl. J. Med. 355, 896–908. doi: 10.1056/NEJMoa060281 16943402

[ref17] Eross L. Halasz L. (2021). Probabilistic Tractography aids Lead placement in Dbs for essential tremor. Stereotact. Funct. Neurosurg. 99, 1–154. doi: 10.1159/000520618

[ref18] Faraji A. H. Kokkinos V. Sweat J. C. Crammond D. J. Richardson R. M. (2020). Robotic-assisted Stereotaxy for deep brain stimulation Lead implantation in awake patients. Operative neurosurgery (Hagerstown, Md) 19, 444–452. doi: 10.1093/ons/opaa029, PMID: 32147722 PMC8223249

[ref19] Fasano A. Romito L. M. Daniele A. Piano C. Zinno M. Bentivoglio A. R. . (2010). Motor and cognitive outcome in patients with Parkinson's disease 8 years after subthalamic implants. Brain J. Neurol. 133, 2664–2676. doi: 10.1093/brain/awq221, PMID: 20802207

[ref20] Fenoy A. J. Simpson R. K. Jr. (2014). Risks of common complications in deep brain stimulation surgery: management and avoidance. J. Neurosurg. 120, 132–139. doi: 10.3171/2013.10.jns131225 24236657

[ref21] Gong S. Tao Y. Jin H. Sun X. Liu Y. Wang S. . (2020). Assessment of deep brain stimulation implantation surgery: a practical scale. World Neurosurg. 134, e1121–e1129. doi: 10.1016/j.wneu.2019.11.117, PMID: 31786379

[ref22] Hamid N. A. Mitchell R. D. Mocroft P. Westby G. W. Milner J. Pall H. (2005). Targeting the subthalamic nucleus for deep brain stimulation: technical approach and fusion of pre- and postoperative Mr images to define accuracy of Lead placement. J. Neurol. Neurosurg. Psychiatry 76, 409–414. doi: 10.1136/jnnp.2003.032029, PMID: 15716537 PMC1739553

[ref23] Hariz G. M. Lindberg M. Bergenheim A. T. (2002). Impact of thalamic deep brain stimulation on disability and health-related quality of life in patients with essential tremor. J. Neurol. Neurosurg. Psychiatry 72, 47–52. doi: 10.1136/jnnp.72.1.47, PMID: 11784825 PMC1737710

[ref24] Hegde A. Canty M. Littlechild P. (2023). A comparison of robotic versus conventional frame based stereotactic Lead placement in Parkinson's disease. Neuromodulation 26:S38. doi: 10.1016/j.neurom.2023.02.061

[ref25] Herzog J. Volkmann J. Krack P. Kopper F. Pötter M. Lorenz D. . (2003). Two-year follow-up of subthalamic deep brain stimulation in Parkinson's disease. Movement disorders: official J Movement Disorder Society 18, 1332–1337. doi: 10.1002/mds.10518, PMID: 14639676

[ref26] Ho A. L. Pendharkar A. V. Brewster R. Martinez D. L. Jaffe R. A. Xu L. W. . (2019). Frameless robot-assisted deep brain stimulation surgery: an initial experience. Operative Neurosurgery 17, 424–431. doi: 10.1093/ons/opy395, PMID: 30629245

[ref27] Ho A. H. Pendharkar A. Brewster R. Parker J. Miller K. Halpern C. (2018). Robot-guided Stereotaxy for deep brain stimulation surgery: an initial experience. J. Neurosurg. 128, 1–84. doi: 10.3171/2018.4.JNS.AANS2018abstracts 30629245

[ref28] Holewijn R. A. Verbaan D. van den Munckhof P. M. Bot M. Geurtsen G. J. Dijk J. M. . (2021). General anesthesia vs local anesthesia in microelectrode recording-guided deep-brain stimulation for Parkinson disease: the galaxy randomized clinical trial. JAMA Neurol. 78, 1212–1219. doi: 10.1001/jamaneurol.2021.2979, PMID: 34491267 PMC8424530

[ref29] Holl E. M. Petersen E. A. Foltynie T. Martinez-Torres I. Limousin P. Hariz M. I. . (2010). Improving targeting in image-guided frame-based deep brain stimulation. Neurosurgery 67, ons437–ons447. doi: 10.1227/NEU.0b013e3181f7422a, PMID: 21099570

[ref30] Holloway K. L. Gaede S. E. Starr P. A. Rosenow J. M. Ramakrishnan V. Henderson J. M. (2005). Frameless Stereotaxy using bone fiducial markers for deep brain stimulation. J. Neurosurg. 103, 404–413. doi: 10.3171/jns.2005.103.3.0404, PMID: 16235670

[ref31] Jankovic J. (2008). Parkinson's disease: clinical features and diagnosis. J. Neurol. Neurosurg. Psychiatry 79, 368–376. doi: 10.1136/jnnp.2007.131045 18344392

[ref32] Jin H. Gong S. Tao Y. Huo H. Sun X. Song D. . (2020). A comparative study of asleep and awake deep brain stimulation robot-assisted surgery for Parkinson's disease. NPJ Parkinson's disease 6:27. doi: 10.1038/s41531-020-00130-1, PMID: 33083521 PMC7536209

[ref33] Joseph J. R. Smith B. W. Liu X. Park P. (2017). Current applications of robotics in spine surgery: a systematic review of the literature. Neurosurg. Focus. 42:E2. doi: 10.3171/2017.2.focus16544, PMID: 28463618

[ref34] Keller D. L. (2013). Neurostimulation for Parkinson's disease with early motor complications. N. Engl. J. Med. 368, 2037–2038. doi: 10.1056/NEJMc1303485, PMID: 23697522

[ref35] Kelman C. Ramakrishnan V. Davies A. Holloway K. (2010). Analysis of stereotactic accuracy of the Cosman-Robert-Wells frame and Nexframe frameless Systems in Deep Brain Stimulation Surgery. Stereotact. Funct. Neurosurg. 88, 288–295. doi: 10.1159/000316761 20588080

[ref36] Khan F. R. Henderson J. M. (2013). Deep brain stimulation surgical techniques. Handb. Clin. Neurol. 116, 27–37. doi: 10.1016/b978-0-444-53497-2.00003-6 24112882

[ref37] Krack P. Batir A. Van Blercom N. Chabardes S. Fraix V. Ardouin C. . (2003). Five-year follow-up of bilateral stimulation of the subthalamic nucleus in advanced Parkinson's disease. N. Engl. J. Med. 349, 1925–1934. doi: 10.1056/NEJMoa035275, PMID: 14614167

[ref38] Krack P. Volkmann J. Tinkhauser G. Deuschl G. (2019). Deep brain stimulation in movement disorders: from experimental surgery to evidence-based therapy. Movement disorders: official J Movement Disorder Society 34, 1795–1810. doi: 10.1002/mds.27860, PMID: 31580535

[ref39] Lake W. Hedera P. Konrad P. (2019). Deep brain stimulation for treatment of tremor. Neurosurg. Clin. N. Am. 30, 147–159. doi: 10.1016/j.nec.2019.01.002 30898267

[ref40] Lanotte M. M. Rizzone M. Bergamasco B. Faccani G. Melcarne A. Lopiano L. (2002). Deep brain stimulation of the subthalamic nucleus: anatomical, neurophysiological, and outcome correlations with the effects of stimulation. J. Neurol. Neurosurg. Psychiatry 72, 53–58. doi: 10.1136/jnnp.72.1.53, PMID: 11784826 PMC1737677

[ref41] Lefranc M. Le Gars D. (2012). Robotic implantation of deep brain stimulation leads, assisted by intra-operative. Flat-Panel Ct. Acta Neurochirurgica 154, 2069–2074. doi: 10.1007/s00701-012-1445-7, PMID: 22814648

[ref42] Lefranc M. Zouitina Y. Tir M. Merle P. Ouendo M. Constans J. M. . (2017). Asleep robot-assisted surgery for the implantation of subthalamic electrodes provides the same clinical improvement and therapeutic window as awake surgery. World Neurosurg. 106, 602–608. doi: 10.1016/j.wneu.2017.07.047, PMID: 28735132

[ref43] Li Y. A comparative study of the precision of deep brain electrical stimulation with surgical robotic assistance and stereotactic framework. Master's thesis of Henan University. (2023).

[ref44] Liang A. S. Ginalis E. E. Jani R. Hargreaves E. L. Danish S. F. (2022). Frameless robotic-assisted deep brain stimulation with the Mazor renaissance system. Operative Neurosurgery 22, 158–164. doi: 10.1227/ons.0000000000000050, PMID: 35166717

[ref45] Limousin P. Pollak P. Benazzouz A. Hoffmann D. Le Bas J. F. Broussolle E. . (1995). Effect of parkinsonian signs and symptoms of bilateral subthalamic nucleus stimulation. Lancet (London, England) 345, 91–95. doi: 10.1016/s0140-6736(95)90062-4 7815888

[ref46] Liu L. Mariani S. G. De Schlichting E. Grand S. Lefranc M. Seigneuret E. . (2020). Frameless Rosa® robot-assisted Lead implantation for deep brain stimulation: technique and accuracy. Operative neurosurgery (Hagerstown, Md) 19, 57–64. doi: 10.1093/ons/opz320, PMID: 31647105

[ref47] Matias C. M. Frizon L. A. Nagel S. J. Lobel D. A. Machado A. G. (2018). Deep brain stimulation outcomes in patients implanted under general anesthesia with frame-based Stereotaxy and intraoperative Mri. J. Neurosurg. 129, 1572–1578. doi: 10.3171/2017.7.jns171166, PMID: 29372880

[ref48] McClelland S. 3rd Ford B. Senatus P. B. Winfield L. M. Du Y. E. Pullman S. L. . (2005). Subthalamic stimulation for Parkinson disease: determination of electrode location necessary for clinical efficacy. Neurosurg. Focus. 19, E12–E19. doi: 10.3171/foc.2005.19.5.13, PMID: 16398462

[ref49] Mei S. Yu K. Ren Z. Hu Y. Guo S. Li Y. . (2022). Techniques of frameless robot-assisted deep brain stimulation and accuracy compared with the frame-based technique. Brain Sci. 12, 1–11. doi: 10.3390/brainsci12070906, PMID: 35884713 PMC9313029

[ref50] Mian M. K. Campos M. Sheth S. A. Eskandar E. N. (2010). Deep brain stimulation for obsessive-compulsive disorder: past, present, and future. Neurosurg. Focus. 29:E10. doi: 10.3171/2010.4.focus10107, PMID: 20672912

[ref51] Moran C. H. Pietrzyk M. Sarangmat N. Gerard C. S. Barua N. Ashida R. . (2020). Clinical outcome of "asleep" deep brain stimulation for Parkinson disease using robot-assisted delivery and anatomic targeting of the subthalamic nucleus: a series of 152 patients. Neurosurgery 88, 165–173. doi: 10.1093/neuros/nyaa367 32985669

[ref52] Moran C. Sarangmat N. Gerard C. S. Barua N. Ashida R. Woolley M. . (2020). Two hundred twenty-six consecutive deep brain stimulation electrodes placed using an "asleep" technique and the neuro|Matetm robot for the treatment of movement disorders. Operative neurosurgery (Hagerstown, Md) 19, 530–538. doi: 10.1093/ons/opaa176, PMID: 32629477

[ref53] Neudorfer C. Hunsche S. Hellmich M. El Majdoub F. Maarouf M. (2018). Comparative study of robot-assisted versus conventional frame-based deep brain stimulation stereotactic neurosurgery. Stereotact. Funct. Neurosurg. 96, 327–334. doi: 10.1159/000494736, PMID: 30481770

[ref54] Ostrem J. L. Galifianakis N. B. Markun L. C. Grace J. K. Martin A. J. Starr P. A. . (2013). Clinical outcomes of Pd patients having bilateral Stn Dbs using high-field interventional Mr-imaging for Lead placement. Clin. Neurol. Neurosurg. 115, 708–712. doi: 10.1016/j.clineuro.2012.08.019, PMID: 22944465 PMC3578021

[ref55] Paff M. Wang A. S. Phielipp N. Vadera S. Morenkova A. Hermanowicz N. . (2020). Two-year clinical outcomes associated with robotic-assisted subthalamic Lead implantation in patients with Parkinson's disease. J. Robot. Surg. 14, 559–565. doi: 10.1007/s11701-019-01025-x, PMID: 31520275

[ref56] Page M. J. McKenzie J. E. Bossuyt P. M. Boutron I. Hoffmann T. C. Mulrow C. D. . (2021). The Prisma 2020 statement: an updated guideline for reporting systematic reviews. BMJ (Clinical research ed) 372:n71. doi: 10.1136/bmj.n71, PMID: 33782057 PMC8005924

[ref57] Qiu Chang. Improvement of deep brain stimulation surgical technical guide by multimodal imaging and electrophysiology. Master's thesis, Nanjing Medical University. (2019).

[ref58] Rahman M. Murad G. J. Mocco J. (2009). Early history of the stereotactic apparatus in neurosurgery. Neurosurg. Focus. 27:E12. doi: 10.3171/2009.7.focus09118 19722814

[ref59] Ribault S. Simon E. Berthiller J. Polo G. Nunes A. Brinzeu A. . (2021). Comparison of clinical outcomes and accuracy of electrode placement between robot-assisted and conventional deep brain stimulation of the subthalamic nucleus: a single-center study. Acta Neurochir. 163, 1327–1333. doi: 10.1007/s00701-021-04790-7, PMID: 33649878

[ref60] Rodriguez-Oroz M. C. Obeso J. A. Lang A. E. Houeto J. L. Pollak P. Rehncrona S. . (2005). Bilateral deep brain stimulation in Parkinson's disease: a multicentre study with 4 years follow-up. Brain J. Neurol. 128, 2240–2249. doi: 10.1093/brain/awh571, PMID: 15975946

[ref61] Sato K. Aita N. Hokari Y. Kitahara E. Tani M. Izawa N. . (2019). Balance and gait improvements of postoperative rehabilitation in patients with Parkinson's disease treated with subthalamic nucleus deep brain stimulation (Stn-Dbs). Parkinson's disease 2019:7104071. doi: 10.1155/2019/7104071, PMID: 31467660 PMC6701295

[ref62] Schulder M. Mishra A. Mammis A. Horn A. Boutet A. Blomstedt P. . (2023). Advances in technical aspects of deep brain stimulation surgery. Stereotact. Funct. Neurosurg. 101, 112–134. doi: 10.1159/000529040, PMID: 36809747 PMC10184879

[ref63] Sharma M. Rhiew R. Deogaonkar M. Rezai A. Boulis N. (2014). Accuracy and precision of targeting using frameless stereotactic system in deep brain stimulator implantation surgery. Neurol. India 62, 503–509. doi: 10.4103/0028-3886.144442, PMID: 25387619

[ref64] Slim K. Nini E. Forestier D. Kwiatkowski F. Panis Y. Chipponi J. (2003). Methodological index for non-randomized studies (Minors): development and validation of a new instrument. ANZ J. Surg. 73, 712–716. doi: 10.1046/j.1445-2197.2003.02748.x, PMID: 12956787

[ref65] Soler-Rico M. Peeters J.-B. Joris V. Delavallee M. Duprez T. Raftopoulos C. (2022). Mri-guided Dbs of Stn under general anesthesia for Parkinson's disease: results and microlesion effect analysis. Acta Neurochir. 164, 2279–2286. doi: 10.1007/s00701-022-05302-x, PMID: 35841433

[ref66] Stang A. (2010). Critical evaluation of the Newcastle-Ottawa scale for the assessment of the quality of nonrandomized studies in Meta-analyses. Eur. J. Epidemiol. 25, 603–605. doi: 10.1007/s10654-010-9491-z, PMID: 20652370

[ref67] Starr P. A. Martin A. J. Ostrem J. L. Talke P. Levesque N. Larson P. S. (2010). Subthalamic nucleus deep brain stimulator placement using high-field interventional magnetic resonance imaging and a skull-mounted aiming device: technique and application accuracy. J. Neurosurg. 112, 479–490. doi: 10.3171/2009.6.jns081161, PMID: 19681683 PMC2866526

[ref68] Starr P. A. Turner R. S. Rau G. Lindsey N. Heath S. Volz M. . (2004). Microelectrode-guided implantation of deep brain stimulators into the Globus pallidus internus for dystonia: techniques, electrode locations, and outcomes. Neurosurg. Focus. 17, 20–31. doi: 10.3171/foc.2004.17.1.4 15264773

[ref69] Stieglitz L. H. Fichtner J. Andres R. Schucht P. Krähenbühl A. K. Raabe A. . (2013). The silent loss of Neuronavigation accuracy: a systematic retrospective analysis of factors influencing the mismatch of frameless stereotactic Systems in Cranial Neurosurgery. Neurosurgery 72, 796–807. doi: 10.1227/NEU.0b013e318287072d 23334280

[ref70] Tolleson C. Stroh J. Ehrenfeld J. Neimat J. Konrad P. Phibbs F. (2014). The factors involved in deep brain stimulation infection: a large case series. Stereotact. Funct. Neurosurg. 92, 227–233. doi: 10.1159/000362934, PMID: 25096381

[ref71] Vadera S. Chan A. Lo T. Gill A. Morenkova A. Phielipp N. M. . (2017). Frameless stereotactic robot-assisted subthalamic nucleus deep brain stimulation: case report. World Neurosurg. 97, 762.e11–762.e14. doi: 10.1016/j.wneu.2015.11.009, PMID: 26585721

[ref72] VanSickle D. Volk V. Freeman P. Henry J. Baldwin M. Fitzpatrick C. K. (2019). Electrode placement accuracy in robot-assisted asleep deep brain stimulation. Ann. Biomed. Eng. 47, 1212–1222. doi: 10.1007/s10439-019-02230-3 30796551

[ref73] von Langsdorff D. Paquis P. Fontaine D. (2015). In vivo measurement of the frame-based application accuracy of the Neuromate neurosurgical robot. J. Neurosurg. 122, 191–194. doi: 10.3171/2014.9.jns14256, PMID: 25361490

[ref74] Weaver F. M. Follett K. Stern M. Hur K. Harris C. Marks W. J. Jr. . (2009). Bilateral deep brain stimulation vs best medical therapy for patients with advanced Parkinson disease: a randomized controlled trial. JAMA 301, 63–73. doi: 10.1001/jama.2008.929, PMID: 19126811 PMC2814800

[ref75] Wu W. D. Gong S. Lei W. Wang S. M. Huang B. H. Yuan L. J. . (2023). The efficacy analysis of neurosurgical robot-assisted Dbs in the treatment of elderly Parkinson's disease. Zhonghua Yi Xue Za Zhi 103, 3816–3821. doi: 10.3760/cma.j.cn112137-20231006-00642, PMID: 38123222

[ref76] Xu F. Jin H. Yang X. Sun X. Wang Y. Xu M. . (2018). Improved accuracy using a modified registration method of Rosa in deep brain stimulation surgery. Neurosurg. Focus. 45:E18. doi: 10.3171/2018.4.focus1815, PMID: 30064312

[ref77] Zangiabadi N. Diana Ladino L. Sina A. Pablo Orozco-Hernandez J. Carter A. Tellez-Zenteno J. F. (2019). Deep brain stimulation and drug-resistant epilepsy: a review of the literature. Front. Neurol. 10:10. doi: 10.3389/fneur.2019.00601 31244761 PMC6563690

[ref78] Zhang J. Application and basic research of stereotactic technology with frame in Parkinson's disease. Doctoral dissertation of Nanchang University. (2020).

