# Supplementary material for: Efficacy and safety of robot-assisted deep brain stimulation for Parkinson’s disease: a meta-analysis
Source: Front Aging Neurosci. 2024 May 31;16:1419152. doi: 10.3389/fnagi.2024.1419152 (PMC11176545; doi:10.3389/fnagi.2024.1419152)
Supplement: Supplementary file 1 [file Table_1.DOCX]

| PubMed | | |
| --- | --- | --- |
| No. | Query | Results |
| 1 | (((((((((((((((((((Parkinson Disease, Idiopathic[Title/Abstract]) OR (Parkinson's Disease, Lewy Body[Title/Abstract])) OR (Parkinsonism, Primary[Title/Abstract])) OR (Parkinson's Disease, Idiopathic[Title/Abstract])) OR (Idiopathic Parkinson Disease[Title/Abstract])) OR (Idiopathic Parkinson's Disease[Title/Abstract])) OR (Lewy Body Parkinson's Disease[Title/Abstract])) OR (Parkinson's Disease[Title/Abstract])) OR (idiopathic parkinsonism[Title/Abstract])) OR (Lewy bodies of Parkinson disease[Title/Abstract])) OR (Lewy bodies of Parkinson`s disease[Title/Abstract])) OR (Lewy bodies of Parkinsons disease[Title/Abstract])) OR (Lewy body Parkinson disease[Title/Abstract])) OR (Lewy body Parkinson`s disease[Title/Abstract])) OR (Lewy body Parkinsons disease[Title/Abstract])) OR (paralysis agitans[Title/Abstract])) OR (Parkinson dementia complex[Title/Abstract])) OR (Parkinsons disease[Title/Abstract])) OR (primary parkinsonism[Title/Abstract])) OR (Parkinson disease[Title/Abstract]) | 124615 |
| 2 | ((((((((((((Brain Stimulations, Deep[Title/Abstract]) OR (Deep Brain Stimulations[Title/Abstract])) OR (Stimulation, Deep Brain[Title/Abstract])) OR (Stimulations, Deep Brain[Title/Abstract])) OR (Brain Stimulation, Deep[Title/Abstract])) OR (Electrical Stimulation of the Brain[Title/Abstract])) OR (brain excitation[Title/Abstract])) OR (brain stimulation[Title/Abstract])) OR (brain stimulus[Title/Abstract])) OR (deep brain stimulation[Title/Abstract])) OR (electrical brain stimulation[Title/Abstract])) OR (excitation, brain[Title/Abstract])) OR (brain depth stimulation[Title/Abstract]) | 41512 |
| 3 | robot[Title/Abstract] OR robotic[Title/Abstract] | 66573 |
| 4 | randomized controlled tria[Title/Abstract] OR controlled clinical trial[Title/Abstract] OR randomized[Title/Abstract] OR placebo[Title/Abstract] OR clinical trials as topic[Title/Abstract] OR randomly[Title/Abstract] OR trial[Title/Abstract] | 1561596 |
| 5 | 1 AND 2 AND 3 | 28 |

| Cochrane library | | |
| --- | --- | --- |
| No. | Query | Results |
| 1 | (Parkinson Disease, Idiopathic OR Parkinson's Disease, Lewy Body OR Parkinsonism, Primary OR Parkinson's Disease, Idiopathic OR Idiopathic Parkinson Disease OR Idiopathic Parkinson's Disease OR Lewy Body Parkinson's Disease OR Parkinson's Disease OR idiopathic parkinsonism OR Lewy bodies of Parkinson disease OR Lewy bodies of Parkinson`s disease OR Lewy bodies of Parkinsons disease OR Lewy body Parkinson disease OR Lewy body Parkinson`s disease OR Lewy body Parkinsons disease OR paralysis agitans OR Parkinson dementia complex OR Parkinsons disease OR primary parkinsonism OR Parkinson disease):ti,ab,kw | 13061 |
| 2 | (Brain Stimulations, Deep OR Deep Brain Stimulations OR Stimulation, Deep Brain OR Stimulations, Deep Brain OR Brain Stimulation, Deep OR Electrical Stimulation of the BrainOR brain excitation OR brain stimulation OR brain stimulus OR deep brain stimulation OR electrical brain stimulation OR excitation, brain OR brain depth stimulation):ti,ab,kw | 14403 |
| 3 | (robot OR robotic):ti,ab,kw | 7205 |
| 4 | (randomized controlled tria OR controlled clinical trial OR randomized OR placebo OR clinical trials as topic OR randomly OR trial):ti,ab,kw | 1508977 |
| 5 | 1 AND 2 AND 3 | 11 |

| Web of science | | |
| --- | --- | --- |
| No. | Query | Results |
| 1 | TS=(Parkinson Disease, Idiopathic OR Parkinson's Disease, Lewy Body OR Parkinsonism, Primary OR Parkinson's Disease, Idiopathic OR Idiopathic Parkinson Disease OR Idiopathic Parkinson's Disease OR Lewy Body Parkinson's Disease OR Parkinson's Disease OR idiopathic parkinsonism OR Lewy bodies of Parkinson disease OR Lewy bodies of Parkinson`s disease OR Lewy bodies of Parkinsons disease OR Lewy body Parkinson disease OR Lewy body Parkinson`s disease OR Lewy body Parkinsons disease OR paralysis agitans OR Parkinson dementia complex OR Parkinsons disease OR primary parkinsonism OR Parkinson disease) | 232250 |
| 2 | TS=(Brain Stimulations, Deep OR Deep Brain Stimulations OR Stimulation, Deep Brain OR Stimulations, Deep Brain OR Brain Stimulation, Deep OR Electrical Stimulation of the BrainOR brain excitation OR brain stimulation OR brain stimulus OR deep brain stimulation OR electrical brain stimulation OR excitation, brain OR brain depth stimulation) | 219566 |
| 3 | TS=(robot OR robotic) | 217855 |
| 4 | TS=(randomized controlled tria OR controlled clinical trial OR randomized OR placebo OR clinical trials as topic OR randomly OR trial) | 3214676 |
| 5 | 1 AND 2 AND 3 | 116 |

| Embase | | |
| --- | --- | --- |
| No. | Query | Results |
| 1 | 'Parkinson Disease, Idiopathic':ab,ti OR 'Parkinson`s Disease, Lewy Body ':ab,ti OR 'Parkinsonism, Primary':ab,ti OR 'Parkinson`s Disease, Idiopathic':ab,ti OR 'Idiopathic Parkinson Disease':ab,ti OR 'Idiopathic Parkinson`s Disease':ab,ti OR 'Lewy Body Parkinson`s Disease':ab,ti OR 'Parkinson`s Disease':ab,ti OR 'idiopathic parkinsonism':ab,ti OR 'Lewy bodies of Parkinson disease':ab,ti OR 'Lewy bodies of Parkinson`s disease':ab,ti OR 'Lewy bodies of Parkinsons disease':ab,ti OR 'Lewy body Parkinson disease':ab,ti OR 'Lewy body Parkinson`s disease':ab,ti OR 'Lewy body Parkinsons disease':ab,ti OR 'paralysis agitans':ab,ti OR 'Parkinson dementia complex':ab,ti OR 'Parkinsons disease':ab,ti OR 'primary parkinsonism':ab,ti OR 'Parkinson disease':ab,ti | 175242 |
| 2 | 'Brain Stimulations, Deep':ab,ti OR 'Deep Brain Stimulations':ab,ti OR 'Stimulation, Deep Brain':ab,ti OR 'Stimulations, Deep Brain':ab,ti OR 'Brain Stimulation, Deep':ab,ti OR 'Electrical Stimulation of the Brain':ab,ti OR 'brain excitation':ab,ti OR 'brain stimulation':ab,ti OR 'brain stimulus':ab,ti OR 'deep brain stimulation':ab,ti OR 'electrical brain stimulation':ab,ti OR 'excitation, brain':ab,ti OR 'brain depth stimulation':ab,ti | 33903 |
| 3 | robot:ab,ti OR robotic:ab,ti | 98013 |
| 4 | 'randomized controlled trial':ab,ti OR 'controlled clinical trial':ab,ti OR randomized:ab,ti OR placebo:ab,ti OR 'clinical trials as topic':ab,ti OR randomly:ab,ti OR trial:ab,ti | 2226299 |
| 5 | 1 AND 2 AND 3 | 43 |
